# Rest-Activity Rhythm Differences in Acute Rehabilitation Between Poststroke Patients and Non–Brain Disease Controls: Comparative Study

**DOI:** 10.2196/49530

**Published:** 2024-07-04

**Authors:** Huey-Wen Liang, Chueh-Hung Wu, Chen Lin, Hsiang-Chih Chang, Yu-Hsuan Lin, Shao-Yu Chen, Wei-Chen Hsu

**Affiliations:** 1 Department of Physical Medicine and Rehabilitation National Taiwan University Hospital Hsin-Chu Branch Hsinchu Taiwan; 2 Department of Physical Medicine and Rehabilitation College of Medicine, National Taiwan University Taipei Taiwan; 3 Department of Physical Medicine and Rehabilitation National Taiwan University Hospital Taipei Taiwan; 4 Department of Biomedical Sciences and Engineering National Central University Taoyuan City Taiwan; 5 Institute of Population Health Sciences National Health Research Institutes Miaoli County Taiwan; 6 Department of Psychiatry National Taiwan University Hospital Taipei Taiwan; 7 Department of Psychiatry College of Medicine National Taiwan University Taipei Taiwan

**Keywords:** circadian rhythms, stroke rehabilitation, rest-activity rhythms, relative amplitude, delirium screening, interdaily stability

## Abstract

**Background:**

Circadian rhythm disruptions are a common concern for poststroke patients undergoing rehabilitation and might negatively impact their functional outcomes.

**Objective:**

Our research aimed to uncover unique patterns and disruptions specific to poststroke rehabilitation patients and identify potential differences in specific rest-activity rhythm indicators when compared to inpatient controls with non–brain-related lesions, such as patients with spinal cord injuries.

**Methods:**

We obtained a 7-day recording with a wearable actigraphy device from 25 poststroke patients (n=9, 36% women; median age 56, IQR 46-71) and 25 age- and gender-matched inpatient control participants (n=15, 60% women; median age 57, IQR 46.5-68.5). To assess circadian rhythm, we used a nonparametric method to calculate key rest-activity rhythm indicators—relative amplitude, interdaily stability, and intradaily variability. Relative amplitude, quantifying rest-activity rhythm amplitude while considering daily variations and unbalanced amplitudes, was calculated as the ratio of the difference between the most active 10 continuous hours and the least active 5 continuous hours to the sum of these 10 and 5 continuous hours. We also examined the clinical correlations between rest-activity rhythm indicators and delirium screening tools, such as the 4 A’s Test and the Barthel Index, which assess delirium and activities of daily living.

**Results:**

Patients who had a stroke had higher least active 5-hour values compared to the control group (median 4.29, IQR 2.88-6.49 vs median 1.84, IQR 0.67-4.34; *P*=.008). The most active 10-hour values showed no significant differences between the groups (stroke group: median 38.92, IQR 14.60-40.87; control group: median 31.18, IQR 18.02-46.84; *P*=.93). The stroke group presented a lower relative amplitude compared to the control group (median 0.74, IQR 0.57-0.85 vs median 0.88, IQR 0.71-0.96; *P*=.009). Further analysis revealed no significant differences in other rest-activity rhythm metrics between the two groups. Among the patients who had a stroke, a negative correlation was observed between the 4 A’s Test scores and relative amplitude (ρ=–0.41; *P*=.045). Across all participants, positive correlations emerged between the Barthel Index scores and both interdaily stability (ρ=0.34; *P*=.02) and the most active 10-hour value (ρ=0.42; *P*=.002).

**Conclusions:**

This study highlights the relevance of circadian rhythm disruptions in poststroke rehabilitation and provides insights into potential diagnostic and prognostic implications for rest-activity rhythm indicators as digital biomarkers.

## Introduction

Sleep-wake cycle disruptions are prevalent in patients who had a stroke and are marked by disturbances in sleep architecture and circadian rhythms [1,2]. These disruptions often lead to fragmented sleep patterns and increase nighttime wakefulness. Poor sleep quality negatively impacts neuroplasticity and memory consolidation post stroke [3,4], potentially affecting recovery outcomes. Additionally, it correlates with reduced daily motor function [5], diminishes physical activity levels, and increases fatigue during inpatient rehabilitation [6]. Higher levels of sleep disruption are tied to a slower recovery of functional independence and motor skills during inpatient rehabilitation [7,8].

During hospital stays, sleep-wake cycle disturbances are frequent, often because of clinical care interventions and a noisy environment [9]. Such impacts are particularly detrimental to stroke survivors, hampering neurological recovery and draining the energy necessary for optimal rehabilitation. Despite the high prevalence of sleep-wake cycle in patients who had a stroke, many questions about the predictors of these disorders, their link to stroke characteristics and severity, and their progression over time remain [10]. Sleep-wake disturbances encompass various factors crucial to the rehabilitation process, such as activity levels, sleep-wake cycles, rest-activity rhythms (RARs), and delirium. These factors often lead to severe circadian rhythm disruptions [11,12], which could directly and negatively influence rehabilitation outcomes by diminishing engagement and impairing learning and consolidation.

Neurocognitive disorders, mainly delirium and dementia [13], represent significant challenges within the realm of poststroke rehabilitation, primarily due to their shared core manifestation of cognitive dysfunction often coupled with disturbances in the sleep-wake cycle. Although dementia typically progresses slowly and over an extended period, delirium presents with an acute onset, making their management particularly complex in the context of stroke recovery. The significance of addressing dementia and delirium in poststroke rehabilitation becomes evident when considering their profound impact on the recovery process. Delirium, with an occurrence rate ranging from 21% to 32% in patients with acute stroke [14], poses a considerable hurdle to rehabilitation efforts. Defined by the *Diagnostic and Statistical Manual of Mental Disorders, Fifth Edition* [13] as an acute and fluctuating disturbance of attention, cognition, or consciousness, delirium not only complicates the clinical picture but also predicts a poor functional outcome for patients who have a stroke [15]. The diagnostic challenge lies in distinguishing delirium from preexisting neurocognitive disorders, underscoring the importance of early identification. The coexistence of sleep-wake disturbance with delirium further exacerbates the situation, emphasizing the critical need for vigilant monitoring and management strategies. Using tools such as actigraphy, which effectively documents altered patterns of motor activity or immobility [16-18], presents an opportunity for early detection and intervention [19]. Despite its proven utility in various clinical settings, its application in patients who had a stroke and experienced delirium remains underexplored [20], highlighting a gap in current research. Moreover, the pioneering work of Witting et al in 1990 laid the foundation for analyzing RARs in neurodegenerative disorders, including dementia [21]. By quantifying parameters such as interdaily stability (IS) and intradaily variability (IV), their framework provided valuable insights into the relationship between RARs and aging-related cognitive decline. This expanded interest in RARs underscores the importance of considering sleep-wake disturbances in the context of dementia and delirium within poststroke rehabilitation.

However, evaluations of sleep-wake disturbances have not been comprehensive within the clinical framework. Most studies primarily focused on sleep and traditionally depended on subjective assessments, such as self-reported questionnaires, daily sleep records, or observations by health care professionals. Objective assessments like polysomnography were also used. Yet these methods did not characterize the activity levels throughout the entire day, which would reflect the full impact of sleep-wake cycle disturbances. Recent advancements in wearable technology have made continuous and noninvasive monitoring of physiological outputs, including motor activity, possible. Ambulatory motor activity recordings, such as RARs based on actigraphy, have proven instrumental in understanding the changes in daily rhythms in patients who had a stroke [11,22,23] and in assessing stroke risk and poststroke outcomes [24]. Considering the well-documented long-term adverse outcomes in poststroke survivors, including a heightened risk of disability and mortality, grasping the relationship between altered RARs and these outcomes is essential for long-term management of post-stroke recovery and rehabilitation [25,26].

While past research has explored the sleep-wake cycle for patients undergoing rehabilitation post stroke, most studies have compared them to age- and gender-matched control groups of healthy community-dwelling adults [11,12]. This design did not account for potential confounding factors like significantly reduced physical activity levels and the influences of hospitalization and acute illness. Therefore, a gap exists in the literature concerning research that included inpatient control participants, especially those with reduced mobility.

Both poststroke patients and those with non–brain-related conditions who were admitted for rehabilitation likely experienced circadian rhythm disruptions. Given that patients who had a stroke faced added challenges from brain injuries, specific RAR indicators were expected when they were compared to inpatient controls with non–brain-related lesions, such as those with spinal cord or orthopedic injuries. This study mainly focused on poststroke rehabilitation patients, compared with the age-matched control group consisting of individuals without brain injuries undergoing rehabilitation. Both groups likely experienced similar reductions in physical activity levels during rehabilitation, providing a controlled setting to investigate RAR indicators specific to poststroke rehabilitation. The primary goal was to analyze RAR indicators between these groups, emphasizing patterns unique to poststroke patients. Additionally, the aim was to correlate RAR indicators with activities of daily living (ADL) and delirium screening tools, ultimately validating the RAR indicators as potential digital biomarkers with diagnostic and prognostic implications.

## Methods

### Study Design and Participants

This study was part of a prospective investigation into the changes and impacts of circadian rhythm and depression on the functional outcomes for patients who had a stroke. We recruited participants from rehabilitation wards in 2 affiliated hospitals of a university in northern Taiwan, with admissions occurring between February and August 2023. They were invited within the first week following their admission to the rehabilitation wards. To qualify, participants of the stroke group needed to be aged ≥20 years, with a confirmed diagnosis of stroke by brain computerized tomography or magnetic resonance imaging, and had been admitted for intensive rehabilitation. The criteria for inclusion in the inpatient control group were being aged ≥20 years and having been admitted for intensive rehabilitation for diagnoses other than brain conditions. Both groups were required to be medically and neurologically stable and to have been healthy and independent before the current admission. Common exclusion criteria for both groups included the following: having an acute illness or unstable vital signs, having an underlying terminal disease, having previous brain conditions (eg, Parkinson disease, stroke, or dementia), being pregnant, and being unable or refusing to use a wearable device for 7 days. Inpatient control participants were age-matched (±3 years) with the stroke group participants.

A power analysis conducted using G*Power (version 3.1; Heinrich-Heine University) was used to determine the required sample size to compare the two groups. The Type I error rate, denoted as alpha (α), was set to a value of .05, while the power was set to 0.95 for a 2-tailed comparison. A total sample size of 50 (25 per group) was required according to the mean (SD) obtained from a previous study using an actigraphy device to evaluate circadian rhythm [27].

### Ethical Considerations

The study adhered to the Declaration of Helsinki, and the protocol received approval from the ethical committee of the National Taiwan University Hospital on January 11, 2023 (approval number 202212047RINA). All participants had provided written informed consent before participation and were given the opportunity to opt out at any time. They signed consent forms that specifically addressed the use and publication of their data. Regarding privacy and confidentiality, all data were deidentified to ensure the protection of participant information. No additional compensation was provided to the participants for their involvement in this study. This decision was made clear to the participants during the informed consent process, ensuring transparency and fairness in the study’s conduct.

### Data Collection

#### Medical Records

We thoroughly reviewed the medical records of all participants. This review covered their demographic details, previous medical and personal histories, specifics about their current condition leading to rehabilitation, medication history—especially concerning sedatives and hypnotics, and their ADL as measured by the Barthel Index (BI) at the time of admission. The data were retrieved from the nurses’ records as a routine evaluation in the rehabilitation wards. The BI is a commonly used and reliable tool to measure functional disability in neurorehabilitation [28,29]. There are 10 items regarding the level of assistance required for basic ADL and the sphincter control. The total scores range from 0 to 100, with a higher score indicating a higher independence.

For those diagnosed with a stroke, we delved deeper, collecting data about the stroke type (infarction or hemorrhage) and category as proposed by the Oxfordshire Community Stroke Project [30]. We also noted any interventions they underwent during the acute stage, such as the administration of recombinant tissue plasminogen activator or mechanical thrombectomy. A particular focus of our review was on the medication history, emphasizing benzodiazepines and antipsychotics, given their potential sleep disturbances or delirium during the study.

Shortly after participants began wearing the actigraphy device, within the first week, nurses screened patients with stroke for delirium using the 4 A’s Test (4AT). Recognized as a rapid delirium assessment instrument, the 4AT consists of 4 items regarding alertness, cognition, attention, and any sudden changes or fluctuations in these areas [31]. The total score ranges from 0 to 12, and a score of 4 or more suggests delirium. It is not a diagnostic tool, but excellent reliability and validity is established through multiple studies, including among patients who had a stroke [32]. Before the onset of the project, all involved nurses underwent a 30-minute training session to ensure they were familiar with the screening’s purpose and procedures. To ensure the process’s accuracy and adherence to standards, a senior nurse, well versed in the evaluation, observed the initial screenings.

#### Actigraphy and RAR measures

Participants were instructed to wear the research-grade wrist actigraphy device (MiCor A100, MiTAC Inc) for a minimum of 7 days. For patients who had a stroke, the actigraphy device was placed on the unaffected side to minimize the impact of limb weakness. For inpatient controls, it was attached to the nondominant side, which most participants preferred. This placement choice was supported by research suggesting that the specific wrist used for actigraphy does not significantly affect the measurement of key sleep variables [33]. Additionally, it was noted that none of the participants experienced bilateral paralysis. Accelerations along 3 axes were collected by the actigraphy watches, combined using Euclidean distance of the accelerations’ deviation from 0, and bandpass filtered from 0.5-3 Hz. The 0 values exceeding a predefined threshold were integrated within 2 seconds, and the activities of 1-second epochs (acti-counts) were derived from averaging the integrated segments within 1 minute [21,34].

We used nonparametric approaches to quantify RARs. The nonparametric method was used to calculate 3 of the circadian rhythm indicators [35]: relative amplitude (RA), IS, and IV.

RA, the ratio of the differences between the most active 10 continuous hours (M10) and the least active 5 continuous hours (L5) over the summation of M10 and L5, was calculated to measure the amplitude of RARs while considering the daily variations and unbalanced amplitudes of peak and trough in daily activity rhythms, as follows:

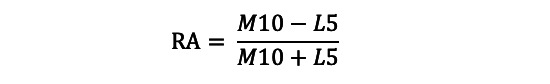



IS quantified the stability of the rhythms between days, that is, the coupling strength of the rhythms with supposedly stable environmental factors. It could vary between 0 and 1, with higher values indicating more stable daily rhythms, as follows:

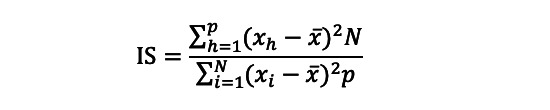



where *N* represents the number of hourly averaged acti-counts, indicating the total data points across all days considered; *p* is the number of data points in the daily circadian template, such as 24 points for an hourly template; *X_h_* denotes the template values at each hour; and *X_i_* refers to the observed values at each hour.

IV indicated the fragmentation of the rhythms, that is, the frequency and extent of transitions between rest and activity. It could vary roughly between 0 and 2, with higher values indicating higher fragmentations, as follows:

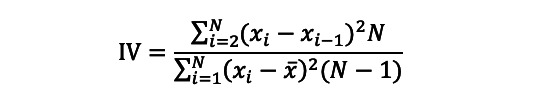



where *N* denotes the total number of hourly averaged acti-counts, and *X_i_* indicates the values observed at each hour.

RA, IS, and IV were calculated for a minimum period of 1 week [35].

#### Physical Activity

The physical activity level of each participant was quantified by using an accelerometer to measure their daily movements. Key features were calculated for each day, including the M10 and L5 values. The M10 and L5 represent the 10 hours of the day when the participant was most active and the 5 hours when they were least active, respectively. These are widely recognized indicators of a person’s circadian activity patterns [36,37]. To determine the M10, a 10-hour moving average was used to estimate the period of the day with the highest average acceleration, which was considered to be the participant’s overall physical activity level.

### Statistical Analysis

The normality of distribution for all circadian parameters derived from the actigraphy was assessed using the Shapiro-Wilk test, although it is noteworthy that in health sciences, most variables do not adhere to a normal distribution due to the nature of the field. Depending on the data distribution, descriptive statistics were either reported as means (SDs) or medians (IQR). To investigate relationships among circadian parameters, BI scores, and 4AT scores, the Spearman ρ correlation coefficient (for nonnormally distributed data) were used. It is important to note that while the Pearson correlation coefficient *r* measures linear relationships in variables following a normal distribution, the use of Spearman ρ is more appropriate for our data set due to its nonnormal distribution. Comparative analyses between the stroke and inpatient control groups, including demographic features, BI scores, 4AT scores, and circadian parameters, were conducted. For nonnormally distributed data, the Mann-Whitney *U* test was used, while the independent 2-tailed *t* test was used for normally distributed data. Furthermore, categorical variables were compared using the chi-square test, with frequencies and percentages reported accordingly.

All statistical computations were executed using SPSS for Windows (version 15.0; IBM Inc). A threshold of *P*<.05 was determined for statistical significance.

## Results

**Figure 1:**
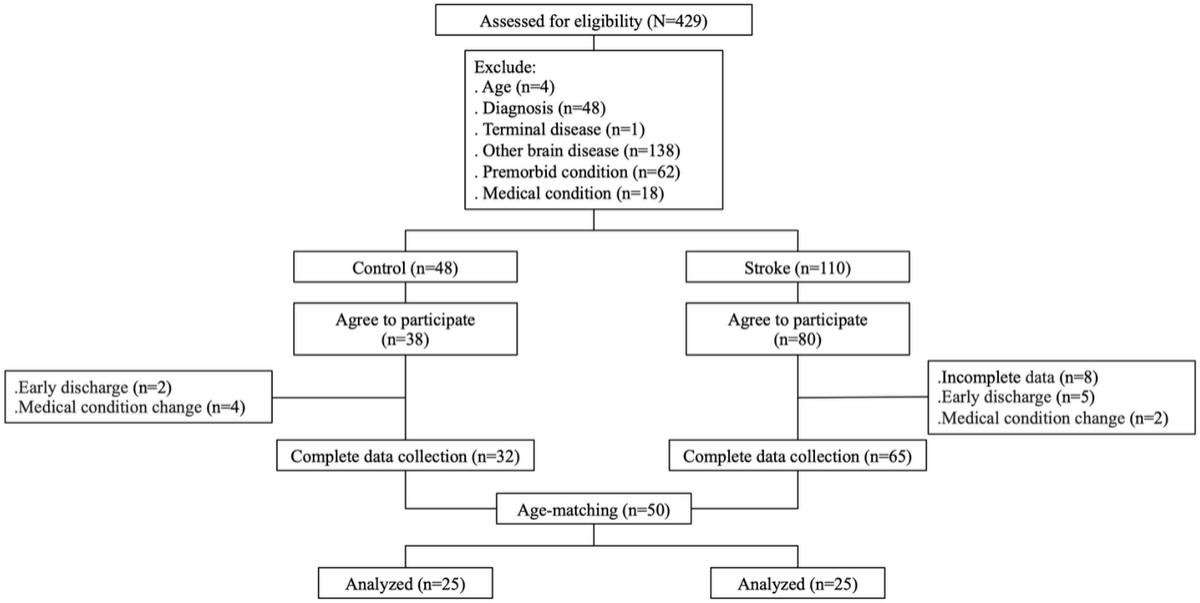
The CONSORT (Consolidated Standards of Reporting Trials) charts of the study participants.

**Figure 2:**
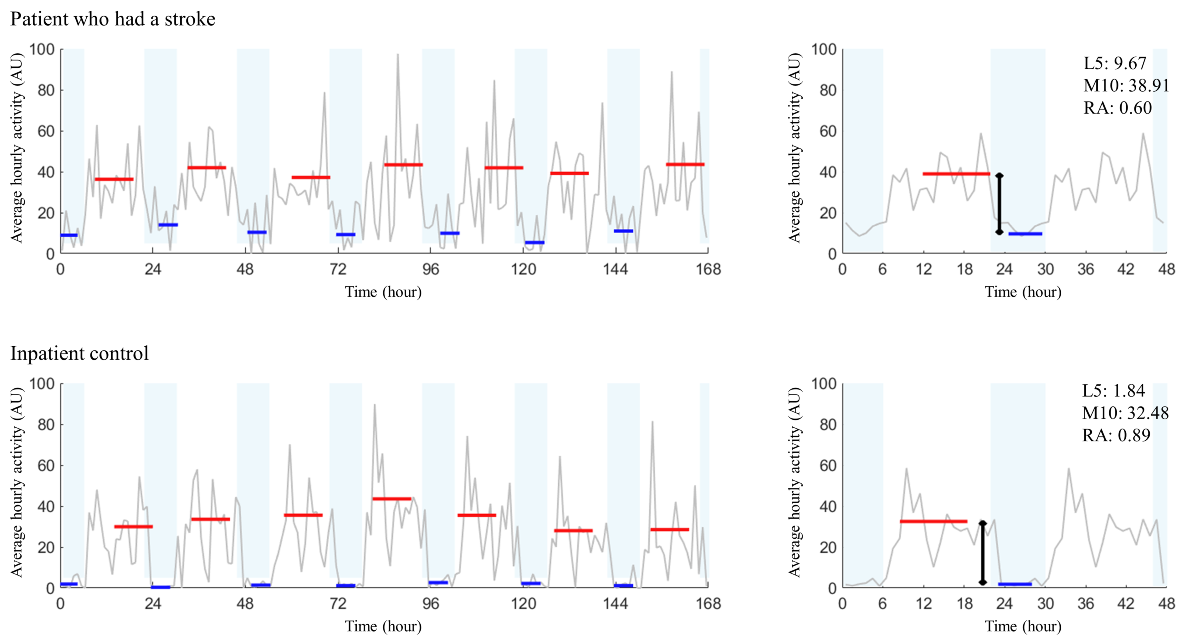
Seven-day rest-activity rhythm analysis of a patient who had a stroke versus an inpatient control. On the left, daily data over the span of a week is presented. Red lines represent the most active 10 continuous hours (M10), while blue lines denote the least active 5 continuous hours (L5), both of which are marked for every 24 hours. On the right, average 7-day data display the composite values of M10, L5, and the relative amplitude (RA), calculated as RA = (M10 – L5) / (M10 + L5). For the stroke group patient, the data are as follows: intradaily variability (IV)=1.55; interdaily stability (IS)=0.57; L5=9.67; M10=38.91; and RA=0.60. In comparison, the inpatient control group presents with the following data: IV=1.05; IS=0.66; L5=1.84; M10=32.48; and RA=0.89.

As depicted in Figure 1, a total of 25 participants from each group were analyzed. Since the variance of the data was mostly unequal between the two groups, nonparametric methods were used for statistical analysis. The primary diagnoses for the inpatient control group consisted of traumatic spinal cord injuries (n=7, 28%), nontraumatic myelopathy (n=12, 48%), and other neurological diseases (n=6, 24%). Figure 2 provides sample recordings from 2 participants of each group. Age, sex, and other demographic parameters were comparable between the groups according to the Mann-Whitney *U* test, chi-square test, or Fisher exact test (Table 1). The stroke group demonstrated a significantly higher prevalence of hypertension and hyperlipidemia. There was no statistically significant difference in the proportion of previous medication history or current medication with sleep medications or antipsychotics (predominantly quetiapine) between the two groups. The average BI scores for both were below 40, indicating pronounced dependence.

**Table 1:** Demographic and medical characteristics of the participants. Numerical data comparisons were conducted using the Mann-Whitney *U* test, and categorical data were analyzed using the chi-square test or Fisher exact test.

Variables			Stroke group (n=25)		Inpatient control group (n=25)		*P* value^a^	
Women, n (%)			9 (36)		15 (60)		.09	
Age (years), median (range)			56 (46-71)		57 (46.5-68.5)		.83	
BMI (kg/m^2^), median (range)			25.2 (23.3-27.5)		23.2 (19.4-26.8)		.11	
Days after admission to hospitals, median (range)			13 (6.5-22)		18 (5-24)		.40	
Days after admission to rehabilitation wards, median (range)			7 (7-7)		7 (7-7)		.42	
**Marital status, n (%)**								.59
	Married	18 (72)		21 (84)				
	Divorced or widowed	2 (8)		1 (4)				
	Unmarried or others	5 (20)		3 (12)				
**Smoking, n (%)**								.89
	Nonsmoker	18 (72)		20 (80)				
	Past smoker	3 (12)		3 (12)				
	Smoker	4 (16)		2 (8)				
**Occupation, n (%)**								>.99
	Retired or no occupation	9 (36)		8 (32)				
	Part-time	2 (8)		2 (8)				
	Full-time	14 (56)		15 (60)				
**Past history, n (%)**								
	Hypertension	17 (68)		7 (28)		<.001		
	Diabetes mellites	4 (16)		3 (12)		.70		
	Hyperlipidemia	11 (44)		4 (16)		.03		
	Sleep medication	4 (16)		5 (20)		>.99		
Barthel Index on admission, median (range)			40 (2.5-55)		35 (20-60)		.55	
Using sleep medication, n (%)			8 (32)		11 (44)		.38	
**Type of stroke, n (%)**								—^b^
	Large-artery atherosclerosis	11 (44)		—				
	Cardioembolism	11 (44)		—				
	Small-vessel occlusion	6 (24)		—				
	Cardioembolism	3 (12)		—				
	Hemorrhage	9 (36)		—				
	Other	3 (12)		—				
**Side of stroke, n (%)**								—
	Right	10 (40)		—				
	Left	11 (44)		—				
	Bilateral	4 (16)		—				
Treated with recombinant tissue plasminogen activator, n (%)			5 (20)		—		—	
Treated with mechanical thrombectomy, n (%)			3 (12)		—		—	
**4A’s Test score**								—
	Total	2 (0-3)		—				
	0	10 (40)		—				
	1-3	12 (48)		—				
	≥4	3 (12)		—				

^a^*P* values were derived from statistical comparisons between poststroke patient and inpatient control groups.

^b^Not available.

An in-depth comparison of RAR indicators between stroke and the control group is detailed in Table 2. The data highlight unique patterns and provide insights into poststroke patients’ circadian rhythm disruptions. Patients who had a stroke exhibited significantly higher L5 values (representing the least active 5 continuous hours) than the control group, with median and IQR values of 4.29 (2.88-6.49) and 1.84 (0.67-4.34), respectively (*P*=.008 as per the Mann-Whitney *U* test). Conversely, M10 values, which encapsulate the most active 10 continuous hours, showed no significant differences between the groups (stroke group: median 38.92, IQR 14.60-40.87; control group: median 31.18, IQR 18.02-46.84; *P*=.93). This infers that peak activity durations were largely uniform across both cohorts. However, despite the congruence in M10 values, the stroke group (median 0.74, IQR 0.57-0.85) displayed a notably lower RA when juxtaposed with the control group (median 0.88, IQR 0.71-0.96; *P*=.009). Further analysis revealed no significant differences in other nonparametric or parametric RAR metrics between the groups.

**Table 2:** Comparative analysis of rest-activity rhythm indicators and physical activity levels between the stroke and inpatient control groups.

Variables	Stroke group (n=25)	Inpatient control group (n=25)	*P* value^a^
Interdaily stability	0.48 (0.38-0.62)	0.54 (0.35-0.69)	.18
Intradaily variability	1.10 (0.85-1.48)	1.19 (0.89-1.52)	.98
Relative amplitude	0.74 (0.57-0.85)	0.88 (0.71-0.96)	.009
Most active 10 continuous hours	38.92 (14.60-40.87)	31.18 (18.02-46.84)	.93
Least active 5 continuous hours	4.29 (2.88-6.49)	1.84 (0.67-4.34)	.008

^a^*P* values were calculated based on statistical comparisons between the stroke patient and inpatient control groups, using the Mann-Whitney *U* test.

Diving deeper into the stroke group patients, a significantly negative correlation was observed between 4AT scores (a delirium metric) and RA, as presented in Table 3 (*ρ*=–0.41; *P*=.045). For all participants, positive correlations emerged between total BI scores, denoting basic ADL independence, and both IS (*ρ*=0.34; *P=*.02) and M10 values (*ρ*=0.42; *P*=.002).

**Table 3:** Spearman correlation analysis between rest-activity rhythm indicators, 4A’s Test (4AT) scores, and Barthel Index scores.

Instrument			Interdaily stability		Intradaily variability		Relative amplitude		M10^a^		L5^b^
Barthel Index											
	ρ	0.34		–0.14		0.19		0.42		–0.01	
	*P* value	.02		.32		.18		.002		.96	
4AT score^c^											
	ρ	–0.32		0.26		–0.41		–0.36		–0.00	
	*P* value	.12		.21		.045		.07		.98	

^a^M10: the most active 10 continuous hours.

^b^L5: the least active 5 continuous hours.

^c^Correlations for the 4AT scores pertain specifically to patients who had a stroke.

## Discussion

### Principal Findings

Our study unveiled a multitude of key findings emphasizing the clinical significance of specific RAR indicators in the context of poststroke rehabilitation. These findings provided valuable insights into the circadian rhythm disruptions experienced by patients who had a stroke during their recovery journey and underscored the need to consider circadian rhythms as a vital aspect of poststroke care. Our research highlighted the clinical relevance of RA in assessing circadian rhythms in patients who had a stroke. The lower RA observed in these patients, in comparison to the inpatient control group, suggested that patients who experienced a stroke exhibited a less pronounced diurnal activity variation, which could negatively predict their overall recovery and functional outcomes. It became apparent that circadian rhythm disruptions, as indicated by RA, had far-reaching implications for the management and assessment of poststroke patients. Additionally, our study explored the clinical implications of RA components, noting that disruptions in circadian patterns during nighttime and daytime significantly impacted patients’ cognitive function and ADL. In particular, disturbances in nighttime activity patterns were linked to potential sleep disturbances, adversely affecting the overall well-being of these patients. This highlighted the importance of managing these disruptions in comprehensive stroke care. Similarly, robust daytime activity was associated with independence in basic ADL, illustrating the importance of maintaining such patterns to improve functional independence during poststroke rehabilitation.

### Comparison to Prior Research

Compared to prior research, our study represented a significant advancement in our understanding of the clinical significance of specific RAR indicators in poststroke rehabilitation. Our research contributed several key distinctions and valuable insights that enriched our comprehension of circadian rhythm disruptions in patients who had a stroke. Our study’s observations concerning RAR measures, particularly the interplay between RA, L5, and M10, aligned with the findings of earlier large-scale investigations, such as those conducted within the UK Biobank [24]. These preceding studies consistently underscored the pivotal role of circadian disturbances in influencing poststroke outcomes. Significantly, our study reaffirmed these established trends. Moreover, our research extended beyond the scope of a previous study using accelerometer data from the UK Biobank, which encompassed a substantial sample size of 91,105 participants and meticulously controlled for numerous confounding factors [38]. This previous investigation primarily highlighted the association of lower RA with mood disorders, subjective well-being, and cognitive function. However, it did not delve into the specific components of lower RA, particularly how daytime and nighttime activities contributed to the observed associations with well-being and the risk of mood disorders [38]. Our study, on the other hand, delved deeper into the complexities of circadian disruptions and their specific manifestations in patients who had a stroke. We provided a comprehensive comparative analysis between stroke patients and inpatient control groups, focusing on distinct RAR indicators.

In our study, we observed that patients who had a stroke exhibited significantly higher L5 values compared to the control group, which indicated potentially poor sleep quality. This aligns with existing research suggesting that elevated L5 values are associated with sleep fragmentation, adversely affecting motor and cognitive performance [39-42]. Typically, the least active 5 hours in individuals sleeping more than 5 hours should coincide with the main sleep period, and disruptions in this period are reflected in increased L5 values [43]. For instance, in someone who sleeps 7 hours, the 1-hour periods just after falling asleep and before waking might involve some minor movements, but the middle 5 hours, which are usually without movement, are considered the L5 timing. This period represents the L5 value. Thus, an increase in L5 value often corresponds with shorter or more fragmented sleep, particularly during deep sleep phases where increased movement can be detected.

We also considered potential confounding factors, particularly the use of benzodiazepines for sedation and sleep. Although benzodiazepines are typically prescribed to alleviate sleep disturbances and could theoretically serve as indicators of sleep quality [44], we chose not to use them as such in our study due to their complex interplay with delirium and sleep apnea in poststroke patients. Indeed, while benzodiazepines have sedative and muscle-relaxing effects that might theoretically reduce the L5 values and improve sleep quality, their use was common among our study population (poststroke patients: n=8, 42.1%; control group: n=11, 57.9%), yet this did not translate into better sleep quality. This is because in patients who had a stroke, benzodiazepines might increase the risk of delirium or disturb the circadian rhythm, potentially leading to higher L5 values by increasing restlessness. Additionally, the high prevalence of sleep apnea in this group [45], causing fragmented sleep patterns and increased nocturnal activity, could also contribute significantly to elevated L5 values. Clinical guidelines recommend against using benzodiazepines for nighttime agitation in delirium, as they can worsen the condition [46]. Furthermore, the muscle-relaxing effects of benzodiazepines can exacerbate sleep apnea [47] in patients who had a stroke, further degrading sleep quality. These considerations underscore the importance of a holistic approach to managing sleep disturbances in stroke rehabilitation, incorporating both pharmacological and nonpharmacological strategies.

In addition to these revelations, our research reinforced the paramount importance of daily activity patterns in the context of poststroke rehabilitation. We identified positive correlations between total scores on the BI, IS, and the M10 value, firmly establishing the connection between circadian rhythm disruptions and functional independence during rehabilitation.

Furthermore, our cross-sectional observational study has provided insights into potential interventions that could be derived from enhancing digital biomarkers associated with circadian rhythms. A key finding is the significant role of RA in indicating circadian disruptions, where patients who had a stroke exhibited lower RA compared to the control group patients, and higher RA correlated with increased delirium scores. Given that RA is calculated as (M10–L5)/(M10+L5), interventions could focus on increasing M10 to enhance daytime activity or decreasing L5 to stabilize nighttime sleep, thus improving day-night distinctions and potentially enhancing daily living functions, as indicated by the positive correlation with the BI.

Moreover, stabilizing daily routines—not only by increasing daytime activities but also by ensuring regularity in day-to-day schedules—could enhance functional outcomes, as suggested by the positive correlation with IS. Lastly, improving sleep stability, which is particularly disrupted in patients who had a stroke (higher L5), could reduce delirium symptoms, further supporting the recovery process. These tailored interventions highlight the importance of precise and targeted approaches to managing circadian rhythm disruptions to improve overall patient outcomes in poststroke recovery.

### Methodological Strengths

Our research design incorporated several methodological strengths that contributed to the significance of our study. First, we focused on circadian disruption, delirium, and other indicators specific to poststroke patients undergoing rehabilitation. While sleep disturbances were well recognized after stroke, our study delved into less explored aspects of sleep quality in this population, shedding light on how these factors may impact recovery and rehabilitation outcomes. Second, unlike many previous studies that used healthy community-dwelling individuals as control groups [11,12], we used a more robust control group design. By comparing poststroke rehabilitation patients with inpatient control participants with non–brain-related lesions undergoing rehabilitation, we accounted for the unique circumstances and challenges faced by both groups during their inpatient stay. This approach enabled us to better isolate the effects of circadian rhythm disruptions from other factors, such as activity levels, environmental effects, and daily routines, which could significantly impact circadian rhythms. Moreover, our study used wearable sensors for monitoring circadian rhythms, aligning with recent advancements in health care technology. The use of wearable sensors provided a low-cost and practical method for monitoring sleep quality in hospital settings. This methodological approach addressed the need for more accessible and cost-effective ways to assess sleep quality in clinical environments, potentially making it easier for health care professionals to incorporate sleep monitoring into routine care. Lastly, our study aligned with recent research trends in the field. We acknowledged the study [20], which explored delirium detection using wearable sensors and machine learning in patients with intracerebral hemorrhage. This study emphasized the growing importance of wearable sensor technology in health care and highlighted the relevance of our approach in the broader context of neurological and rehabilitation research.

### Limitations

Several methodological limitations should be taken into account when interpreting the findings of our study. First, the evaluation of delirium using the 4AT tool was exclusively administered to patients who had a stroke, resulting in a lack of corresponding delirium assessment for the control group. The participants in the control group had no history of brain-related lesions and were not routinely assessed for delirium in the wards. This discrepancy limits our ability to draw direct comparisons between the two groups in terms of delirium incidence. Second, both the stroke patient group and the control group exhibited limited levels of physical activity, making it impractical to apply standard actigraphy algorithms to distinguish sleep and wake states accurately. However, despite these constraints, our study provided valuable insights into sleep quality by analyzing nocturnal activity patterns, with a particular emphasis on L5 values. These observations allowed us to make informed inferences regarding potential sleep fragmentation and suboptimal sleep quality among patients who had a stroke. Finally, our assessment of ADL was based on cross-sectional observations, which may not fully capture the longitudinal dynamics of their relationship with rehabilitation outcomes. A more extended, longitudinal study would be necessary to explore the long-term effects of circadian rhythm disruptions on poststroke recovery comprehensively.

In conclusion, our study illuminated the critical role of specific RAR indicators in poststroke rehabilitation, shedding light on the clinical significance of circadian rhythm disruptions in this context. The lower RA observed in poststroke patients suggested a potential avenue for intervention, indicating that circadian disturbances might be modifiable risk factors for stroke. By addressing these disturbances and promoting robust daytime and nighttime activity patterns aligned with the circadian cycle, health care professionals could potentially enhance stroke rehabilitation and improve the overall quality of poststroke care. Furthermore, our research underscored the emergence of “digital biomarkers” in the field of neurological and rehabilitation research. Wearable sensor technology, as demonstrated in our study, offered a practical and cost-effective means of monitoring circadian rhythms and sleep quality in clinical settings. The integration of digital biomarkers into routine health care assessments holds great promise for enhancing the precision and personalization of patient care, enabling early interventions, and ultimately improving stroke outcomes.

## Abbreviations

ADL: activities of daily living
BI: Barthel Index
IS: interdaily stability
IV: intradaily variability
L5: the least active 5 continuous hours
M10: the most active 10 continuous hours
RA: relative amplitude
RAR: rest-activity rhythm
4AT: 4 A’s Test

## Data Availability

The datasets used and analyzed during this study are available from the corresponding author upon reasonable request.
